# Special Issue: Advanced Biopolymer-Based Nanocomposites and Hybrid Materials

**DOI:** 10.3390/ma14030493

**Published:** 2021-01-21

**Authors:** Armando J. D. Silvestre, Carmen S. R. Freire, Carla Vilela

**Affiliations:** CICECO—Department of Chemistry, Aveiro Institute of Materials, University of Aveiro, 3810-193 Aveiro, Portugal; armsil@ua.pt (A.J.D.S.); cfreire@ua.pt (C.S.R.F.)

The gamut of natural polymers, from polysaccharides to proteins, exhibit peculiar features and multiple functionalities that are being exploited to engineer advanced nanocomposites and hybrid materials. In fact, the growing environmental concerns explain the focus of the increasing scientific activity on both polysaccharides (e.g., cellulose, chitosan, alginate, fucoidan, and hyaluronic acid) and proteins (e.g., silk, collagen, and gelatin), given their remarkable potential for the design of all categories of materials for application in multiple fields, for example, in electronics, energy, environment, biology, and medicine [1,2]. This Special Issue of *Materials*—belonging to the section *Biomaterials*—contains a collection of six research papers about advanced biopolymer-based nanocomposites and hybrid materials. The collected papers made use of biopolymers, such as nanocellulose [3], fucoidan [4], silk fibroin [5,6], hyaluronic acid [6], and lignin [7], in combination with a polyzwitterion [3], metal nanoparticles [4,5], organometallic polymers [8], and a thermoplastic polymer [7], to develop advanced systems for water remediation [3], cancer treatment [4], catalysis, sensing, and energy storage [5], tissue engineering [6,8], and 3D printing [7,8].

The investigation of Vilela et al. [3] demonstrated that zwitterionic nanocomposite membranes comprising bacterial nanocellulose and cross-linked poly(2-methacryloyloxyethyl phosphorylcholine) can be used as tools for water remediation. The combination of the bacterial polysaccharide and the polyzwitterion originated robust nanocomposite adsorbent membranes with high water uptake capacity in different pH media and antimicrobial activity against Gram-positive (*Staphylococcus aureus*) and Gram-negative (*Escherichia coli*) pathogenic bacteria. Furthermore, the nanocellulose-based adsorbent membranes were capable of adsorbing two model ionic organic azo dyes, namely methylene blue and methyl orange, with toxicity towards humans and the environment, thus proving their potential in the context of water remediation.

In a different study, Pinto et al. [4] explored the potential of fucoidan-gold nanosystems for cancer therapy. The authors effectively developed gold nanoparticles (AuNPs) coated with fucoidan via one-minute microwave-assisted synthesis using a fucoidan-enriched fraction extracted from *Fucus vesiculosus* (species of brown algae), which played the simultaneous role of reducing and capping agents. This one-minute synthesis originated monodispersed and spherical fucoidan-AuNPs with antitumoral activity against three human tumor cell lines, namely MNT-1 (pigmented human melanoma cells), HepG2 (human hepatocyte carcinoma), and MG-63 (human osteosarcoma) cell lines. Moreover, the combination between flow cytometry and dark-field imaging confirmed the cellular uptake of the fucoidan-AuNPs by the MG-63 cell line.

As pointed out by Mitropoulos et al. [5], noble metal nanoparticles, namely palladium, platinum, and gold, can be anchored at the surface of silk fibroin (SF) to prepare composite aerogel fibers. The porous silk gels were produced by dissolving the SF in hexafluoro-2-propanol (HFIP), followed by casting in silicon tubes and physical crosslinking with ethanol. These gels were then equilibrated in the noble metal salt solutions, reduced with sodium borohydride, and dried via supercritical technology to produce the porous aerogel fibers coated with noble metal nanoparticles, for application in catalysis, energy storage, and conversion.

Chung et al. [6] delve into the effect of SF on the characteristics of hyaluronic acid (HA)/silk fibroin hydrogels for tissue engineering of nucleus pulposus (NP). The authors concluded that the strain of SF and the weight ratios of SF to HA influenced the rheological properties of the crosslinked silk fibroin/hyaluronic acid hydrogels. Additionally, the biocompatible SF/HA hydrogels favored the differentiation of human bone marrow-derived mesenchymal stem cell (hBMSC) to NP cells, which demonstrated the suitability of these hydrogels in tissue engineering for NP regeneration.

In another study, Balčiūnas et al. [8] investigated the biocompatibility of hybrid organometallic polymers for sub-micron 3D printing via laser two-photon polymerization. The Al and Zr containing hybrid organometallic polymers supported cellular growth to full confluency and promoted collagen synthesis. Therefore, these hybrid organometallic polymers can be an asset in constructing the tissue-engineered grafts of the future.

Finally, Tanase-Opedal et al. [7] manufactured composite filaments by compounding the thermoplastic poly(lactic acid) (PLA) with lignin obtained from Spruce biomass via the soda pulping process. The aromatic natural polymer from forestry biomass acted as a nucleating agent, which promoted further crystallization of PLA, and originated composite materials with antioxidant potential for application in 3D printing.

Overall, the six papers of this Special Issue of *Materials* covered some examples of materials resulting from the combination of natural polymers with distinct partners (e.g., synthetic polymers or metal nanoparticles), and contributed to the development of advanced nanocomposites and hybrid materials for both technological and biomedical applications.
